# Immune subgroup analysis for non-small cell lung cancer may be a good choice for evaluating therapeutic efficacy and prognosis

**DOI:** 10.18632/aging.202941

**Published:** 2021-05-11

**Authors:** Yuan Tian, Jingnan Wang, Qing Wen, Guohai Su, Yuping Sun

**Affiliations:** 1 Department of Oncology, Jinan Central Hospital, Shandong University, Jinan 250013, Shandong, P.R. China; 2 Department of Radiotherapy Oncology, Shandong Provincial Qianfoshan Hospital, Shandong University, Jinan 250014, Shandong, P.R. China; 3 Department of Radiotherapy Oncology, Shandong Provincial Qianfoshan Hospital, The First Hospital Affiliated with Shandong First Medical University, Jinan 250014, Shandong, P.R. China; 4 Jinan Clinical Research Center of Shandong First Medical University, Jinan 250013, Shandong, P.R. China; 5 Department of Cardiovascular Diseases, Jinan Central Hospital Affiliated to Shandong University, Jinan 250013, Shandong, P.R. China; 6 Department of Oncology, Jinan Central Hospital Affiliated to Shandong First Medical University, Jinan 250013, Shandong, P.R. China

**Keywords:** immune subgroup, NSCLC, therapeutic efficacy, prognosis

## Abstract

Due to its effectiveness, cancer immunotherapy has attracted widespread attention from clinicians and scientific researchers. Numerous studies have proven that effective stratification of cancer patients would promote the personalized application of immunotherapy. Therefore, we used the transcriptome data of nearly 1,000 patients with non-small cell lung cancer (NSCLC) to construct a new immune subgroup. We found that the new immune subgroup, named cluster 2, was a mixture of lung adenocarcinoma (LUAD) and lung squamous cell carcinoma (LUSC), and showed poor overall survival, which was further verified in the independent validation set. Immune infiltration correlation analysis showed that the Mast cell type and its status subdivisions had a predictive effect on the prognosis of NSCLC, especially in LUAD. Phenotypic analysis suggested that epithelial-mesenchymal transition (EMT) was positively correlated with immunosuppression, supporting the correlation between tumor phenotype and immune background. Although immune subtypes failed to significantly distinguish the progression-free survival (PFS) of immunotherapy patients, they showed the expected trend; the sample size needs to be further expanded for verification. In addition, some results indicated that the two cancer types, LUAD and LUSC, might require independent analyses.

## INTRODUCTION

Tumor immunotherapy has developed rapidly in recent years. Due to its effectiveness and safety [1, 2], it has attracted widespread attention from researchers and clinicians, especially immunotherapy related to PD-1/PD-L1 inhibitors for lung cancers [3–15]. However, even in patients with the same type of tumor, immunotherapy response rates were quite variable, and in some patients the rates were relatively low [3–15]. According to the corresponding immune indicators, researchers have reported that the stratification of cancer patients was helpful for predicting the patients’ prognosis or immunotherapy response rates [16, 17]. For clear cell renal cell carcinoma, it was reported that the potential interaction between immune phenotype and somatic changes affected the efficacy of PD-1 inhibitors [18, 19]. The correlation between immune subgroup and immunotherapy response was also observed in a study on non-small cell lung cancer (NSCLC) [20]. Based on the aforementioned research [16–20], it was speculated that there may be some inherent correlations between immune subgroup and NSCLC, which affect the response rate of immunotherapy and may further affect the prognosis of patients.

With the development of sequencing technology and bioinformatic analysis methods, a large quantity of cancer-related genome, transcriptome, and immune-related information can be obtained through sequencing or reanalysis of sequencing results [21–24]. Bioinformatic analysis has become a common approach for investigating cancer-related indicators [21–24]. It had been reported that the intermediate immune infiltration cluster (Cluster B) was associated with a worse prognosis independently of known clinicopathological features in breast cancers [25]. In order to clarify the inherent relationship between immune subgroup and NSCLC, bioinformatic analyses and online sequencing results were used to construct a new kind of immune subgroup based on the immune infiltration cluster method [25]. Bioinformatic analyses were completed mainly using the R programming language.

## MATERIALS AND METHODS

### The cancer genome atlas (TCGA) expression profile data

TCGA Pan-Cancer Atlas integrated pan-cancer RNA-seq data was downloaded from the Genomic Data Commons (GDC) website (https://gdc.cancer.gov/about-data/publications/pancanatlas). Based on the disease and sample labels, only samples from the original site of LUAD (n=506) and LUSC (n=482) (*-01A) were extracted. Genes for which the expression was 0 in all samples were removed, and fragments per kilobase of exon model per million mapped reads (FPKM) values were normalized to log2. Only one technical duplication remained.

### Gene expression omnibus (GEO) validation data download and preprocessing

GSE4573 chip data was downloaded from the GEO database, and the tumor samples (HG-U133A platform) were extracted, comprising a total of 130 samples. The chip data was standardized based on the Robust Multichip Average method. The expression value of each gene was calculated based on the relationship between the corresponding probe and the gene. For cases in which a gene corresponded to multiple probes, we chose the mean value of these probes as the expression value of the gene. The sample characteristics of TCGA and GEO are shown in Table 1.

**Table 1:** Basic characteristics of the data included in the study.

	**TCGA_NSCLC (N=996)**	**GSE4573 (N=130)**	**Overall (N=1126)**
**Histology**			
LUAD	509 (51.1%)	0 (0%)	509 (45.2%)
LUSC	487 (48.9%)	130 (100%)	617 (54.8%)
**Stage**			
Stage I	509 (51.1%)	73 (56.2%)	582 (51.7%)
Stage II	277 (27.8%)	34 (26.2%)	311 (27.6%)
Stage III	165 (16.6%)	23 (17.7%)	188 (16.7%)
Stage IV	33 (3.3%)	0 (0%)	33 (2.9%)
Missing	12 (1.2%)	0 (0%)	12 (1.1%)
**Gender**			
Female	401 (40.3%)	48 (36.9%)	449 (39.9%)
Male	595 (59.7%)	82 (63.1%)	677 (60.1%)
**Age (years)**			
Mean (SD)	66.2 (9.34)	67.5 (9.86)	66.4 (9.41)
Median [Min, Max]	67.0 [38.0, 90.0]	68.0 [42.0, 91.0]	67.0 [38.0, 91.0]
Missing	28 (2.8%)	0 (0%)	28 (2.5%)
**Smoking_Pack_years**			
Mean (SD)	47.5 (28.9)	62.7 (43.9)	49.5 (31.6)
Median [Min, Max]	40.0 [0.150, 200]	60.0 [0, 300]	45.0 [0, 300]
Missing	237 (23.8%)	18 (13.8%)	255 (22.6%)

### Identification of immune subtypes based on consensus clustering

770 immune-related genes were collected from the nCounter^®^ PanCancer Immune Profiling Panel, and the correlation matrix was calculated among samples based on their expression. Consensus clustering was implemented based on the R package ConsensusClusterPlus, and the clustering method and distance measurement corresponded to Ward.D2 and Pearson correlation distance, respectively. Based on the change in value of the area under the consistent connected dominating set (CDS) and CDS curve, the number of consistent clusters was determined to be 4. Since there was only one sample in category 4, it was removed in the subsequent analysis.

### Immune cell infiltration ratio and immune-related evaluation score

The infiltration ratio of 22 immune cell types in tumor samples was calculated based on the gene expression deconvolution algorithm CIBERSORT [26]. The expression feature Leukocyte signature Matrix 22 (LM22) of immune cell signature genes was constructed using the CIBERSORT algorithm in advance. This expression matrix contained the expression patterns of 547 genes in 22 types of cells [naive B cells, memory B cells, Plasma cells, CD8 T cells, naive CD4 T cells, resting memory CD4 T cells, activated memory CD4 T cells, follicular helper T cells, regulatory T cells (Tregs), gamma delta T cells, resting NK cells, activated NK cells, Monocytes, M0 Macrophages, M1 Macrophages, M2 Macrophages, resting Dendritic cells, activated Dendritic cells, resting Mast cells, activated Mast cells, Eosinophils, and Neutrophils]. For the normalized expression profile data, we used the default immune cell feature matrix LM22, randomly 100 times to obtain the immune infiltration ratio of each sample. Then, the state of the immune cells was distinguished according to the method used by Thorsson [27]. When calculating and analyzing the differences in the proportion of immune cell infiltration, the proportion of infiltration for each cell type was first standardized by z-score; then, the significance of the difference was calculated based on the Kruskal-Wallis test. Lymphoid and myeloid scores were calculated based on Nanodissect [28].

### Prediction of immune subgroups based on binomial logistic regression

An immune subgroup prediction model was developed based on the binomial logistic regression method, and optimized by L1 regularization (lasso) and full subset regression. The model was validated by shuffle-split with five-fold cross-validation. These five models were integrated into an ensemble model to output the final classification index.

### Survival analysis

Kaplan-Meier graphs were generated using the R package survminer. The Maximum Selection Test (Maximally Selected Log-Rank Statistic, R package survminer::surv_cutpoint) method was used to identify the best cutpoint for high/low infiltration or high/low expression.

## RESULTS

### Constructing a new immune subgroup of NSCLC

**Figure 1:**
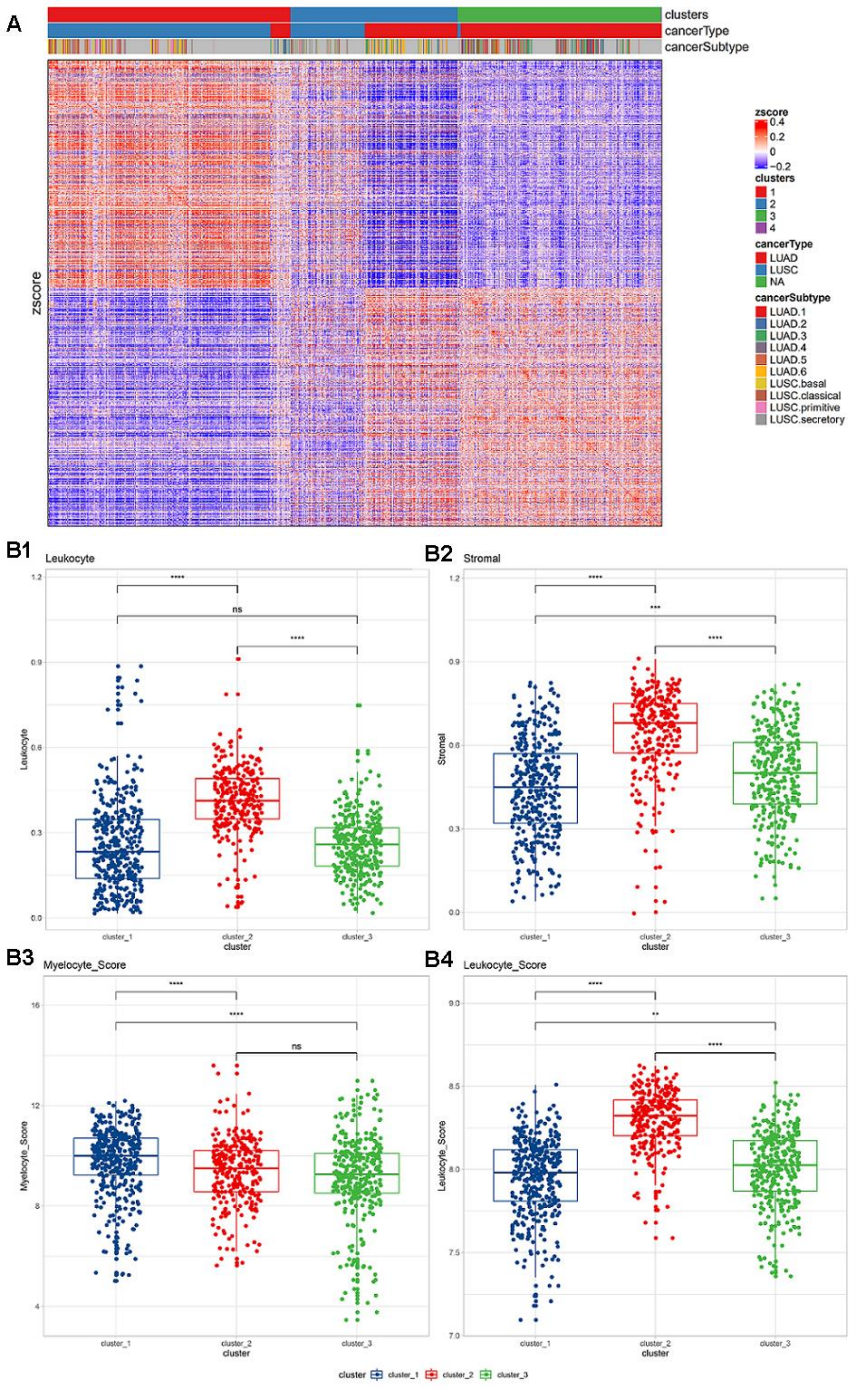
(**A**) Expression of immune-related genes in TCGA non-small cell lung cancer data. Pearson correlation distance and ward.D2 were used for unsupervised clustering. The top annotations represent the subgroups, cancer types, and TCGA cancer subtypes identified based on consistent clustering. (**B**) Lymphocyte and myeloid cell infiltration and stromal cell distribution among subgroups. (**B1**) Lymphocyte infiltration in different clusters; (**B2**) stromal cell distribution in different clusters; (**B3**) myelocyte infiltration score in different clusters; (**B4**) lymphocyte infiltration score in different clusters. The Wilcoxon rank-sum test (*, P<0.05; **, P<0.01; ***, P<0.001; ****, P<0.0001) was used for statistical difference testing among different groups.

The flow diagram is provided in Supplementary Figure 1. Based on the expression levels of 770 immune-related genes derived from the nCounter^®^ Pan-Cancer Immune Profiling array, a consistent clustering of 988 NSCLC samples was put into practice. Based on the change in the area under the consistent CDS and CDS curve, the number of consistent clusters was selected to be 4 (Supplementary Figure 2). Since there was only one sample in category 4, it was removed in the subsequent analysis. In the identified subgroups, most of the samples in cluster 1 (n=394) were squamous carcinomas, almost all of the samples in cluster 3 (n=331) were adenocarcinoma, and the samples in cluster 2 (n=272) were a mixture of the two cancer types (Figure 1A). To confirm that these three clusters were related to the tumor immune microenvironment (TIME), the Nanodissect algorithm was used to score total lymphocytes and myeloid cell infiltration. The three clusters were found to be significantly correlated with the scores of lymphocytes, myeloid cells and stromal cells (Wilcoxon rank-sum test, p<0.0001, Figure 1B).

Based on pathological evaluation, the lymphatic invasion of cluster 2 was the highest and differed significantly from the other two groups, while no difference in lymphatic invasion was found between clusters 1 and 3. The levels of stromal cells in the three subgroups were found to be significantly different; cluster 2 was considered to have the highest level (Figure 1B1, 1B2). Based on the expression level of infiltration score, cluster 2 was also shown with the highest level of lymphatic invasion, while myeloid infiltration was found to be gradually increased in the three subgroups (Figure 1B3, 1B4).

Among the three clusters, the enrichment scores of all immune-related pathways showed significant differences. Most of the pathways showed stronger signal in cluster 2, and were significantly higher than the other two groups (Kruskal test, *, P <0.05; **, P <0.01; ***, P <0.001; ****, P <0.0001; Supplementary Figure 3). Among the three clusters, all suppressive immune checkpoint genes showed significant expression differences. The vast majority of pathways showed significantly high expression in cluster2 (Kruskal test, *, P <0.05; Supplementary Figure 4). Among them, the macrophage CD86 and the widely used ICB target-CD274 are both significantly highly expressed in cluster 2.

### Building an immune subgroup prediction model

**Figure 2:**
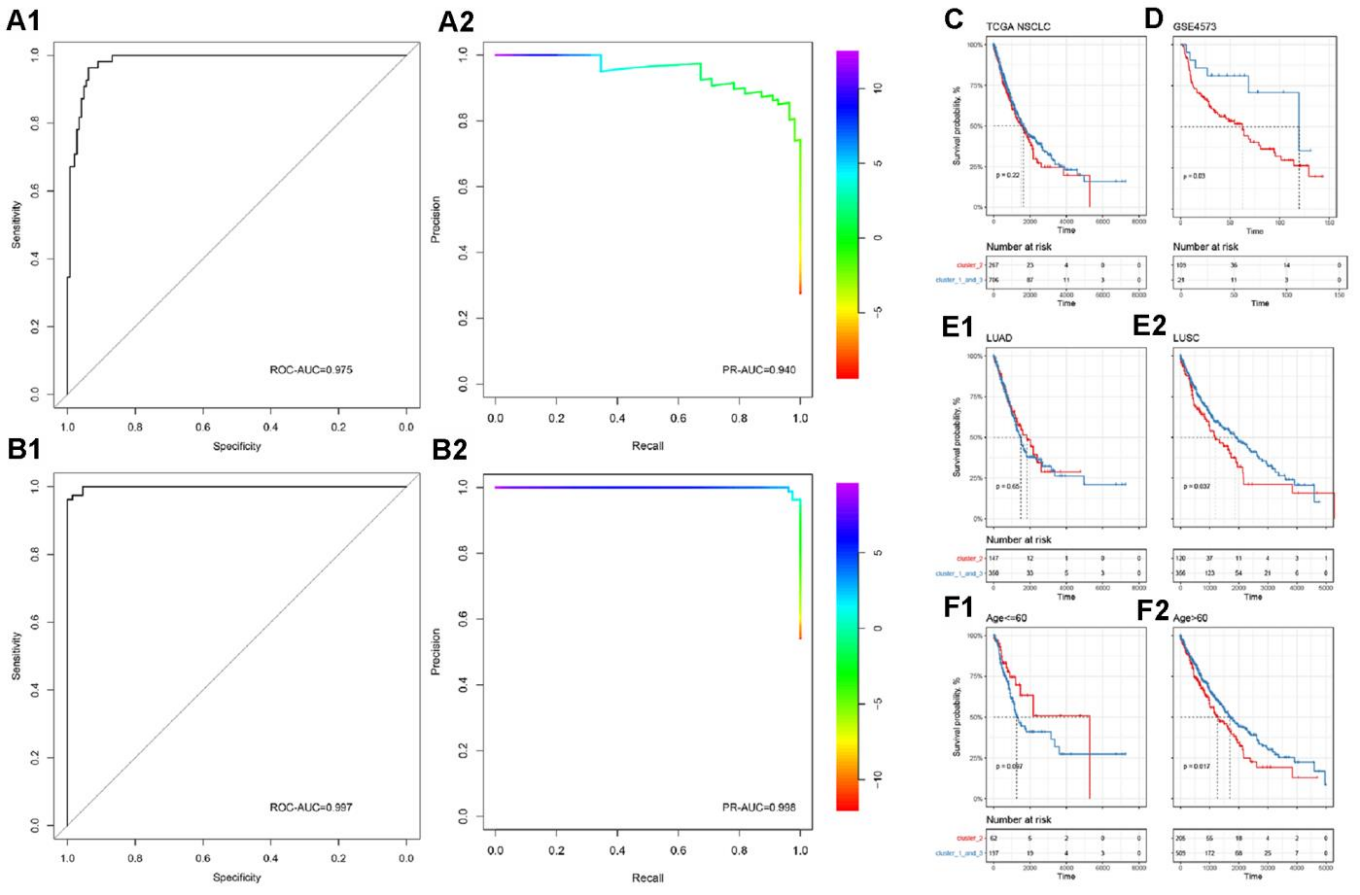
**Subgroup prediction model based on binomial logistic regression and prognostic efficacy of immune subgroups.** (**A**) Distinguishing the predictive power of cluster 2 from the other two groups. (**B**) Distinguishing the predictive power of cluster 1 from cluster 3. The upper part is ROC-AUC, and the lower part is PR-AUC. (**C**) In all NSCLC samples, immune subgroups cannot significantly distinguish the overall survival of patients. (**D**) In the GEO independent verification set, the immune subgroup can significantly distinguish the prognosis of patients. Among patients with lung squamous cell carcinoma (**E**) or those older than 60 years (**F**), immune subgroups can significantly distinguish the prognosis of the patients.

In order to accurately distinguish lung cancer subgroups without relying on unsupervised clustering, we then used binomial logistic regression and L1 regularization (lasso) to construct a subgroup classifier to distinguish cluster 2 from other subgroups (cluster 1 and cluster 3). It was confirmed by five-fold crossover that our model had good predictive power (ROC-AUC=0.975, PR-AUC=0.940, Figure 2A, 2B). Furthermore, we performed a new round of binomial logistic regression to distinguish cluster 1 and cluster 3, which also achieved extremely high performance (ROC-AUC=0.997, PR-AUC=0.998).

### Prognostic efficacy of immune subgroups

To complete our observations, we mapped patient survival based on the identified immune subgroups. Unfortunately, immune subgroup failed to distinguish the overall survival of patients effectively in TCGA-NSCLC (log-rank test, p>0.05, Figure 2C). In the independent validation set, patient survival could be distinguished effectively by the identified immune subgroups (log-rank test, p<0.05, Figure 2D). After considering the effects of age, tumor stage, gender, and cancer type, it was found that the current immune subgroup could significantly distinguish the overall survival from others when patients were over 60 years or had squamous cell carcinoma (log-rank test, p<0.05, Figure 2E, 2F). In addition, progression-free survival (PFS) of Stage II NSCLC patients could also be significantly distinguished by immune subgroup (log-rank test, p<0.05, Supplementary Figure 5).

### Molecular indicators of immune subgroups

**Figure 3:**
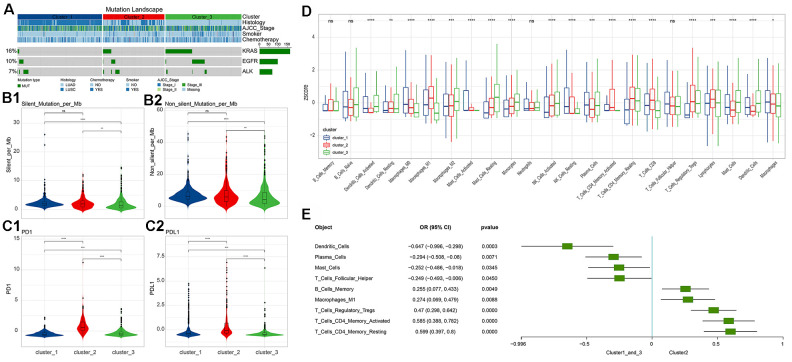
**Differences in molecular indicators among immune subgroups and subtype-related immune cell infiltration.** (**A**) Mutation landscape in all clusters. KRAS and EGFR mutations occurred significantly in cluster 3 (chi square test, p<0.05). (**B**) Tumor mutation burden (Silent and Non-silent) in different clusters. Cluster 3 had the lowest tumor mutation burden. (**C**) Expression levels of PD-1/PD-L1 in different clusters. Cluster 2 had the highest levels of PD-1 and PD-L1 expression. The numerical statistical test was based on the Wilcoxon rank sum test (*, P<0.05; **, P<0.01; ***, P<0.001; ****, P<0.0001). (**D**) Differences in immune cell infiltration subtypes. Statistical significance was calculated using the Kruskal-Wallis test (*, P<0.05; **, P<0.01; ***, P<0.001; ****, P<0.0001). (**E**) Immune cell infiltration significantly associated with cluster 2 (based on logistics regression identification, p<0.05).

Subsequently, we systematically analyzed the molecular and clinical indicators of immune subgroups, including genomic alterations, pathological typing, and immune infiltration. By comparing the three immune subgroups, we found that EGFR and KRAS were significantly different in the subgroups (chi square test, p<0.05, Figure 3A), and mainly appeared in cluster 3. EGFR and KRAS were mutually exclusive in cluster 2 and cluster 3 (chi square test, p<0.05). Analyzing the correlation between the three subgroups and clinical information, we found that smoking was significantly related to immune subgroup, mainly apparent in cluster 3 (chi square test, p<0.05). However, the immune subgroup had nothing to do with American Joint Committee on Cancer (AJCC) Stage or chemotherapy (chi square test, p>0.05). In addition, the silent mutation and non-silent mutation loads of cluster 3 were found to be at the lowest level among the clusters, significantly lower than those of cluster 1 and cluster 2 (Wilcoxon rank-sum test, Figure 3B); there was no significant difference in tumor mutation burden (TMB) between cluster 2 and cluster 1. Turning our attention to PD-1 and PD-L1, we found that the expression levels of these two molecules (z-score of log2-transformed FPKM) in the cluster 2 subgroup were significantly higher than those of the other two subgroups (Wilcoxon rank-sum test, Figure 3C).

### Immune cell infiltration in different subgroups

Based on the CIBERSORT method, we calculated the immune cell infiltration of each sample.

We found that 22 immune cell types and their status subdivisions showed significant differences in the three subgroups. Among them, M1 Macrophages were found to be have the greatest infiltration in cluster 2, while resting Mast cells had the greatest infiltration in cluster 3 (Figure 3D and Supplementary Figure 6). Using the generalized linear model, we identified the immune cell types that could explain the difference between cluster 2 and cluster 1_cluster 3 (Figure 3E).

**Figure 4:**
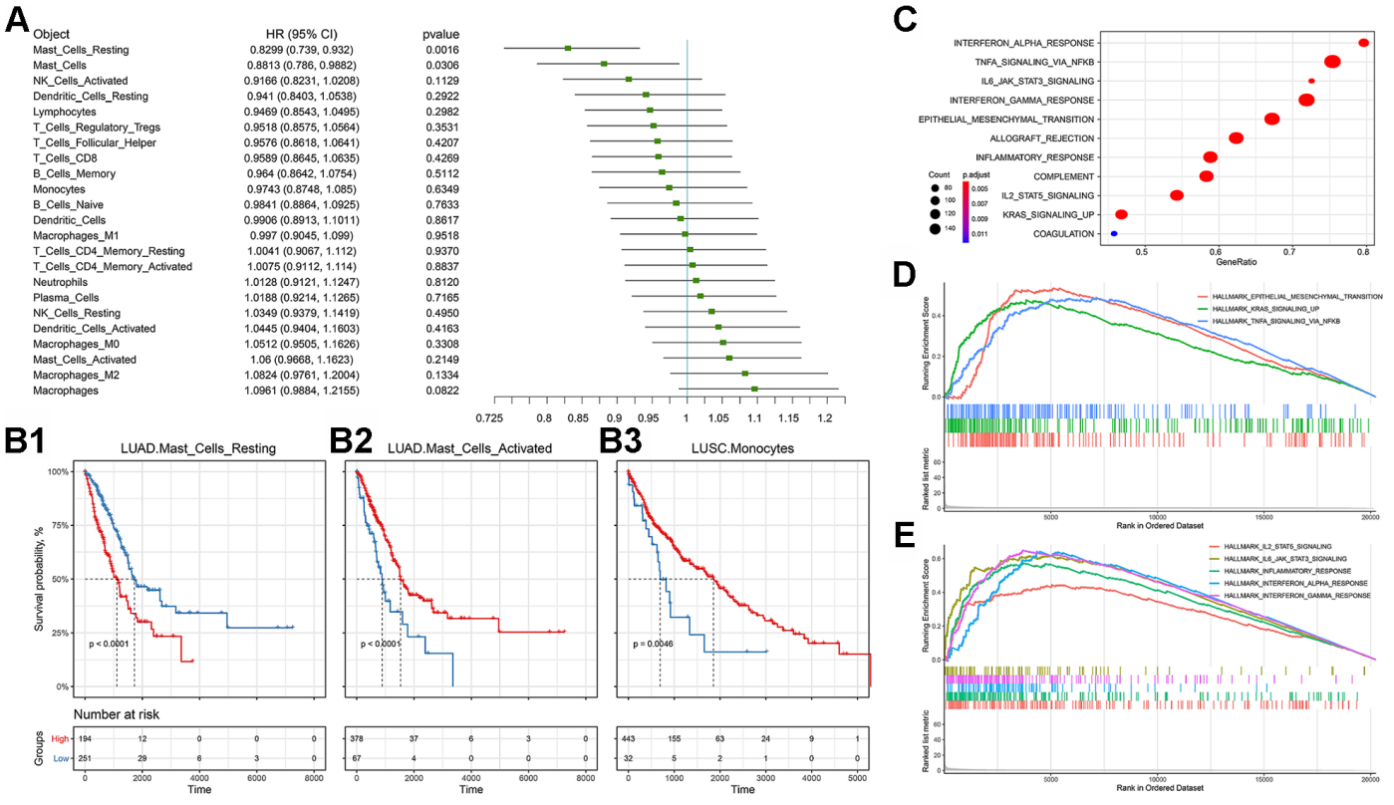
**Prognostic efficacy of immune cell infiltration in TCGA-NSCLC and the functional differences involved in cluster 2.** (**A**) Prognostic efficacy of immune cell infiltration in TCGA-NSCLC: Forest plot of the prognostic efficacy of immune cells. Single factor cox regression was used to evaluate the significance of the correlation between cell infiltration ratio and Overall Survival (OS). (**B**) Prognostic efficacy of immune cell infiltration in TCGA-NSCLC: In the corresponding metabolic subtypes, the Kaplan-Meier curve of the two groups of patients with high and low cell infiltration (Low). The log-rank test was used for calculating the difference in OS between the high and low groups. The division threshold was based on the maximum selection method. (**C**) Functional differences involved in cluster 2: Identifying the functional differences based on the GSEA method. The MSigDB cancer hallmark gene set was used. (**D**) Functional differences involved in cluster 2: Visualization of important cancer-related pathways. (**E**) Functional differences involved in cluster 2: Visualization of important immune-related pathways.

We then observed the correlation between immune cell infiltration and the prognosis of NSCLC, and found that Mast cells and their status subdivisions were significantly associated with the prognosis of patients (Figure 4A). To further subdivide cancer types, we found that the Mast cell type and its status subdivisions could still distinguish the prognosis of patients in LUAD from the others, while Monocytes in LUSC patients was found to be related to the overall survival of patients (Figure 4B). Prior research suggested that the Mast cell type and its phenotype might be related to the prognosis of NSCLC [29]. Furthermore, it was reported that Mast cells might have a prognostic effect on lung cancer [30]. Our results showed that activated and resting Mast cells were significantly associated with better and worse prognoses, respectively, which further supports the prognostic role of the Mast cell type and its status subdivisions in NSCLC. Unlike previous reports, in LUSC, we found that Monocytes were associated with a better prognosis [31–34].

### Functional differences in different subgroups

Based on the MSigDB cancer hallmark gene set, we used the Gene Set Enrichment Analysis (GSEA) method to identify the cluster 2-related functional differences. The results showed that compared with cluster_1_and_3, cluster 2's dysregulated genes were enriched in a variety of pathways related to malignant cancer progression, including stem cell biology and EMT-related pathways corresponding to cancer-related functions (Figure 4C). Further visualizing the important cancer-related pathways, we found that cluster 2 had more EMT, KRAS and TNF signaling pathways, which was consistent with the worse prognosis associated with this cluster (Figure 4D, 4E). At the same time, we found that cluster 2 also had an enriched immune-related factor and active inflammatory response.

**Figure 5:**
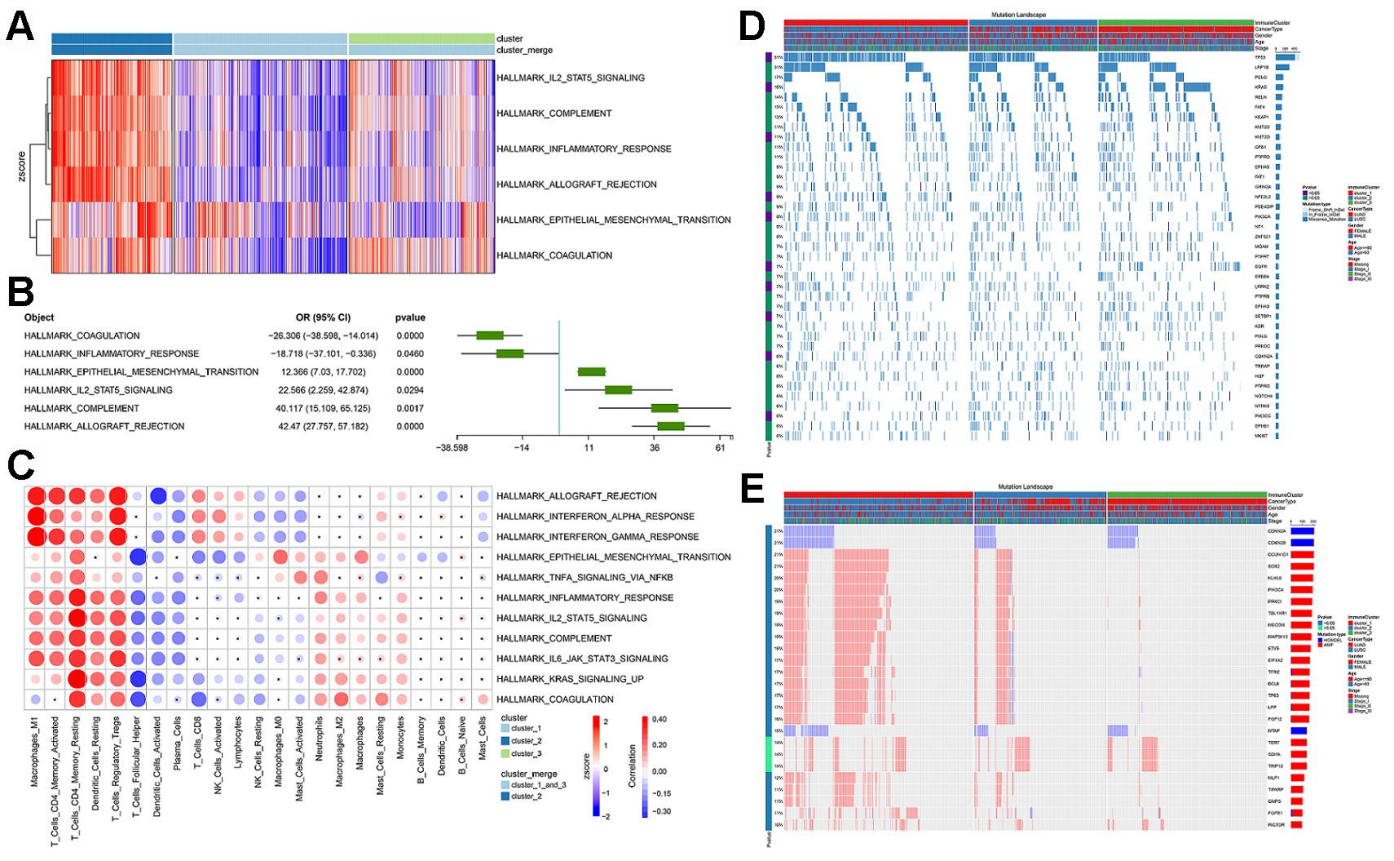
**The functional differences involved in cluster 2 and the correlation between immune infiltration and cluster 2.** (**A**) GSVA score spectrum of functional gene set in cluster 2. (**B**) Application of logistic regression to identify pathways that were significantly related to cluster 2. (**C**) Correlation between cluster function and immune infiltration. (**D**) Gene mutation characteristic blueprint of TCGA-NSCLC immune subgroup. 39 oncogenes mutated in >6% samples in the three immune subgroups of TCGA-NSCLC (missense mutation, dark blue; insert deletion, light blue). The bar graph on the right represents the mutation frequency of each gene in the sample population, and the colored bar graph on the left represents whether the occurrence of the gene mutation was related to the immune subgroup (chi-square test). Each column represents a patient, and the colored bar chart at the top indicates the immune subgroup to which the patient belonged: the subtype of NSCLC, the patient's gender, age group, and tumor grade. (**E**) Copy number variation blueprint of TCGA-NSCLC immune subgroups; the status of 26 oncogenes with copy number changes in >10% samples in the three immune subgroups of TCGA-NSCLC (copy number amplification, red; copy number deletion, blue). The bar graph on the right represents the copy number variation frequency of each gene in the sample population. The colored bar chart on the left represents whether the occurrence of copy number variation of the gene was related to the immune subgroup (chi-square test). Each column represents a patient, and the colored bar chart at the top indicates the immune subgroup to which the patient belonged: the subtype of NSCLC, the patient's gender, age group, and tumor grade.

In order to further explain the relationship between immune subgroup and cancer cell phenotype, we calculated the signature score of the aforementioned gene set based on the Gene Set Variation Analysis (GSVA) method for each sample. This score reflected the activity of each pathway in the immune subgroup. Immune subgroups could be distinguished clearly by unsupervised clustering of gene set scores (Figure 5A). Through the unsupervised clustering of GSVA enrichment scores, we found that those signatures appeared with high scores in cluster 2, but displayed erratic patterns in other subgroups. In order to formally determine which gene set score could explain cluster 2, we used the logistics model to test the contribution of each gene set to cluster 2 and cluster_1_and_3. The results indicate that pathways such as EMT and IL2-STAT5-SIGNALING have a positive contribution to cluster 2, while cluster_1_and_3 is related to inflammatory response (Figure 5B).

Since the immune subgroups were related to a variety of immune-related pathways, we then checked the relationship between immune cell infiltration and the aforementioned cancer-related pathways. We found that the activity scores of these pathways were significantly correlated with infiltration of a variety of immune cells (Pearson’s correlation test, p value<0.05, Figure 5C). It was worth noting that EMT was significantly associated with all immune cells and their status subdivisions. The high EMT score was highly positively correlated with infiltration of a variety of inhibitory immune cells (e.g., M1 Macrophages, resting Dendritic cells, and regulatory T Cells), and negatively correlated with the infiltration of killer immune cells (e.g., CD8 T Cells and Activated NK Cells).

### Subgroup-associated clinical features (EMT and proliferation) mutations, CNV, etc.

We observed the mutation signature blueprints of TCGA-NSCLC samples in three different immune subtypes, and compared the mutations of some key oncogenes (from Oncology Knowledge Base; OncoKB) among different immune subgroups (Figure 5D). Most of the oncogenes with mutation frequency greater than 10% appeared with missense mutations. Among different immune subgroups, the mutations of TP53, KRAS, KMT2D, NFE2L2, PIK3CA, EGFR, LRRK2, SETBP1, CDKN2A, PIK3CG were found to be distributed in a significantly biased manner (Figure 5D) [35, 36]. In TCGA-NSCLC patients, we found that CDKN2A, CDKN2B and MTAP mainly had copy number deletions (Figure 5E), whereas PIK3CA, SOX2, BCL6 had a large number of copy number amplifications and were significantly concentrated in cluster 1.

### Immunotherapy in different subgroups

**Figure 6:**
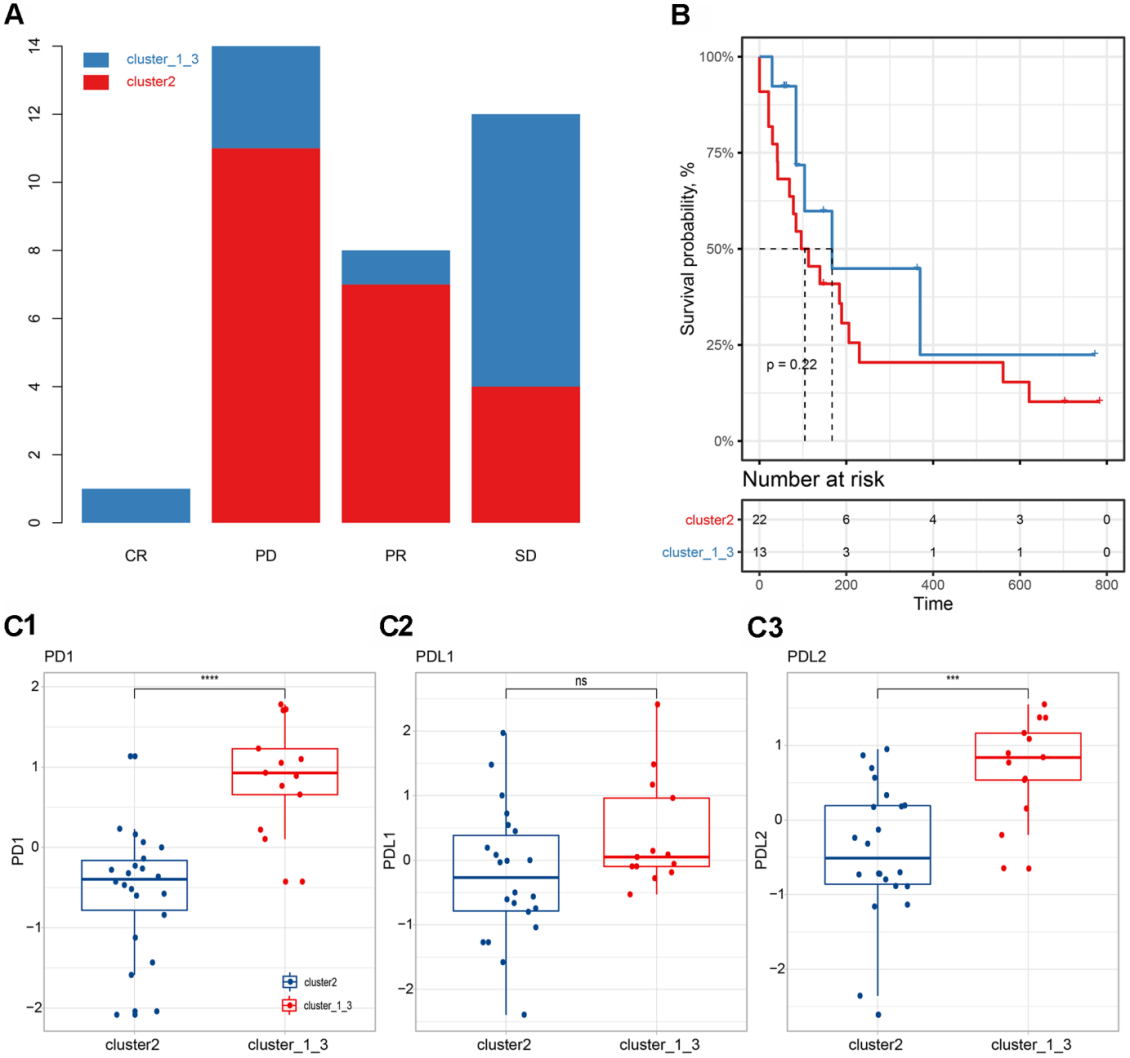
**Correlation between immune subgroups and the efficacy of immunotherapy.** (**A**) Efficacy of immunotherapy in different immune subgroups. (**B**) Progression-free survival in different immune subgroups. (**C**) Expression differences of PD-1 (**C1**), PD-L1 (**C2**) and PD-L2 (**C3**) in different subgroups.

We further evaluated the relationship between immune subgroup and immunotherapy (Figure 6). Based on the previous model, we divided immunotherapy patients into cluster 2 and cluster_1_and_3. We found that 31.4% (11/35) of PD patients, 22.9% (8/35) of PR patients, and 11.4% (4/35) of SD patients belonged to the cluster 2 subgroup, whereas 8.6% (3/35) of PD patients, 2.9% (1/35) of PR patients and 22.9% (8/35) of SD patients belong to the cluster_1_and_3 subgroup. When comparing (CR & PR) vs. PD, there was no significant association between efficacy and immune subgroup (chi square test, p>0.05, Figure 6A). The PFS in the cluster 2 subgroup was shorter than that in the cluster_1_and_3 subgroup, although no significant difference was found (log-rank test, p>0.05, Figure 6B). Observing the expression levels of PD-1, PD-L1, and PD-L2, we found that the expression levels of these genes in the cluster_1_and_3 subgroup were significantly higher than those in the cluster 2 subgroup (Figure 6C).

## DISCUSSION

Among all patients with lung cancer, NSCLC accounts for more than 85% of cases, making it the most common pathological type in clinical work and the leading cause of cancer-related deaths [37]. In recent years, immunotherapy for NSCLC has been developing rapidly, especially for PD-1/PD-L1 inhibitors [38–42]. While tumor immunotherapy has achieved satisfactory anti-tumor efficacy, predictive indicators related to therapeutic efficacy and prognosis have also been gradually reported [16–19, 43]. Based on the aforementioned reports on immune predictive indicators, we designed the present study in order to comprehensively evaluate the impact of immune-related indicators or immune grouping methods on the therapeutic efficacy and prognosis of NSCLC [16–19, 43].

Based on the expression levels of 770 immune-related genes derived from the nCounter^®^ PanCancer Immune Profiling array, 988 NSCLC samples were clustered uniformly, and three cluster subgroups were obtained (Figure 1A), cluster 1 (n=394), cluster 2 (n=272) and cluster 3 (n=331), corresponding to the three most common pathological types in NSCLC: squamous cell carcinoma, adenosquamous cell carcinoma, and adenocarcinoma [44, 45]. We found that these three clusters were significantly correlated with the scores of lymphocytes, myeloid cells and stromal cells (Wilcoxon rank-sum test, p<0.0001, Figure 1B). Based on the Nanodissect algorithm to score total lymphocytes and myeloid cell infiltration (Figure 1B), we inferred that these three clusters may be related to the tumor immune microenvironment. This laid the foundation for us to further construct the immune subgroup prediction model (Figure 2A, 2B).

At present, immune checkpoint inhibitor therapies, especially for PD-1/PD-L1 inhibitors [3–15, 46, 47], have been developing rapidly for NSCLC, and a large number of indicators related to the efficacy of immunotherapy and patient prognosis have also been reported [48–50]. However, the prognostic efficacy of immune subgroups has rarely been reported. After considering the effects of age, tumor stage, gender, and cancer type, we found that overall survival could be significantly distinguished by immune subgroup when patients were over 60 years of age or had squamous cell carcinoma (log-rank test, p<0.05, Figure 2E, 2F). In addition, the PFS survival of Stage II NSCLC patients could also be significantly distinguished by immune subgroup (log-rank test, p<0.05, S Figure 3). In the cluster 2 subgroup, the expression levels of PD-1 and PD-L1 (z-scores of log2-transformed FPKM) were significantly higher than in the other two subgroups (Wilcoxon rank-sum test, Figure 3C). Because the cluster 2 subgroup had higher TMB (Silent mutation per Mb and Non-silent mutation per Mb) and higher PD-1 and PD-L1 expressions, it may be implied to have a better response to immunotherapy [51, 52]. The aforementioned findings would be helpful for us to judge the prognosis of patients in clinical work.

Through the analysis of immune cell infiltration in different subgroups (Figure 3D, 3E), we found that the tumor-promoting immune cell types M1 Macrophages [53], regulatory T Cells [54], activated memory CD4 T Cells [49, 55], and resting memory CD4 T Cells mainly existed in cluster 2, which might explain the reason for the worse prognosis with the cluster 2 subgroup. The analysis results showed that the Mast cell type and its status subdivisions are significantly associated with patient prognosis (Figure 4A). To further subdivide the cancer type, the Mast cell type and its status subdivisions could significantly distinguish the prognosis of patients in LUAD from others, while the presence of Monocytes in LUSC patients was significantly related to the overall survival of these patients (Figure 4B). Our analysis results were similar to those in prior reports [29, 30]. At the same time, the results also verified the feasibility of our analysis method from another aspect. Furthermore, our results showed that activated and resting Mast cells were significantly associated with better and worse prognoses, respectively, which further supports the prognostic role of the Mast cell type and its status subdivisions in NSCLC (Figure 4B1, 4B2). It has been reported that circulating Monocytes are associated with angiogenesis and poor prognosis in numerous cancers [31–34]. In LUSC, we found that Monocytes were associated with a better prognosis (Figure 4B3). This inconsistency may be due to the following reasons: (1) differences caused by the differentiation of monocytes into different cells (mainly including macrophages) after entering tissues; (2) specificity of tissue origin of LUSC.

Through our analysis, we found that more active pathways related to malignant progression of cancer were enriched in the cluster 2 subgroup (Figure 4C), which was consistent with the worse prognosis of this subgroup (Figure 4D). At the same time, we found that cluster 2 also has a strong immune-related factor and inflammatory response (Figure 4E). However, TCGA-NSCLC data are basically chemotherapy related. Therefore, the relationship between this strong immune response and the worse prognosis cannot be explained clearly.

In order to further explain the relationship between immune subgroups and cancer cell phenotype, we calculated the signature score of the aforementioned gene set based on the GSVA method for each sample. Immune subgroups could be distinguished clearly by unsupervised clustering of gene set scores (Figure 5A). The analysis results indicated that cluster 2 was positively influenced by pathways such as EMT and IL2-STAT5-SIGNALING, whereas cluster_1_and_3 was related to inflammatory response (Figure 5B). Furthermore, we found that the activity scores of these pathways were significantly correlated with infiltration of a variety of immune cells (Figure 5C). Notably, EMT was significantly associated with all immune cells and their status subdivisions. EMT score was highly positively correlated with infiltration of a variety of inhibitory immune cells (e.g., M1 Macrophages, resting Dendritic cells, and regulatory T Cells; Figure 5C), and negatively correlated with the infiltration of killer immune cells (e.g., CD8 T Cells and activated NK cells; Figure 5C). This may further explain the poor prognosis of patients in the cluster 2 subgroup [56, 57].

We observed the mutation signature blueprints of TCGA-NSCLC samples in three different immune subtypes, and compared the mutations of some key oncogenes (from OncoKB) among different immune subgroups (Figure 5D, 5E). The mutation frequency of TP53 was as high as 51%, which tended to appear in clusters 1 and 2, while cluster 3 had less mutation enrichment [58, 59]. As a rare oncogene related to the potential treatment of NSCLC, PIK3CA also showed a similar tendency for mutation enrichment, with more mutations in cluster 1 and 2 samples. It was reported that PIK3CA mutations could confer a relapse-free survival advantage for squamous cell carcinoma in NSCLC [35]. On the contrary, EGFR and KRAS mutations were abundantly enriched in cluster 3 samples, which was consistent with the function of suppressing immune infiltration as reported in some studies [36]. In our results, we also observed that the samples in cluster 3 had lower lymphocyte infiltration (Figure 1B). Both the enrichment of mutant genes and the alterations in copy number of related genes were basically consistent with previous reports [35, 36, 60, 61]. In other words, the use of our analysis method will make it easier for clinicians to judge the immune infiltration characteristics, mutations and prognostic survival of tumor patients, hence facilitating the formulation of treatment plans.

We further evaluated the relationship between immune subgroups and immunotherapy (Figure 6). The PFS in cluster 2 subgroups was shorter than those of cluster_1_and_3 subgroups, although no significant difference was found (log-rank test, p>0.05, Figure 6B). Observing the expression levels of PD-1, PD-L1, and PD-L2, we found that the expression levels of these genes in the cluster_1_and_3 subgroup were significantly higher than those in the cluster 2 group (Figure 6C), which might partly explain the slightly better PFS of the cluster_1_and_3 subgroup [51].

## CONCLUSIONS

The immune infiltration cluster 2 subgroup was a mixture of LUAD and LUSC, and showed poor overall survival, which was further verified in the independent validation set. Immune infiltration correlation analysis showed that the Mast cell type and its status subdivisions had a predictive effect on the prognosis of NSCLC, especially in LUAD.

### Statement of ethics

Since our study is based on online public database resources with no interaction with human subjects, no issues related to medical ethics are reported.

## Supplementary Material

Supplementary Figures
