# Supplementary material for: Immune subgroup analysis for non-small cell lung cancer may be a good choice for evaluating therapeutic efficacy and prognosis
Source: Aging (Albany NY). 2021 May 11;13(9):12691–709. doi: 10.18632/aging.202941 (PMC8148502; doi:10.18632/aging.202941)
Supplement: Supplementary Figures [file aging-13-202941-s001.pdf]

SUPPLEMENTARY FIGURES

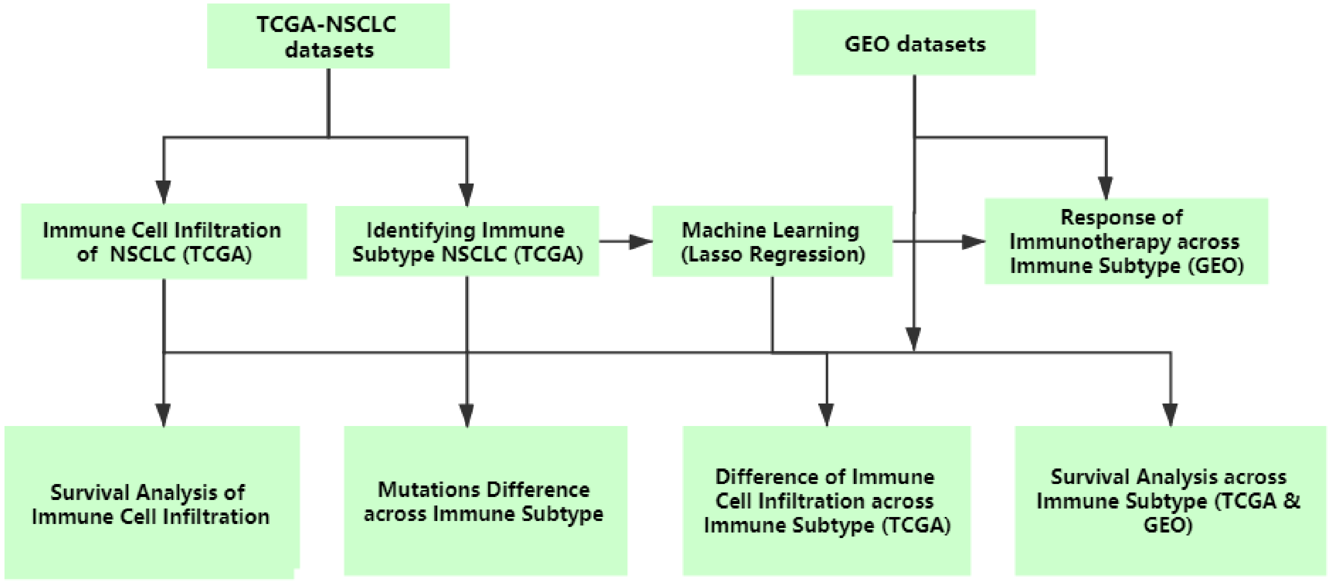

Supplementary Figure 1. Flow diagram of the study.

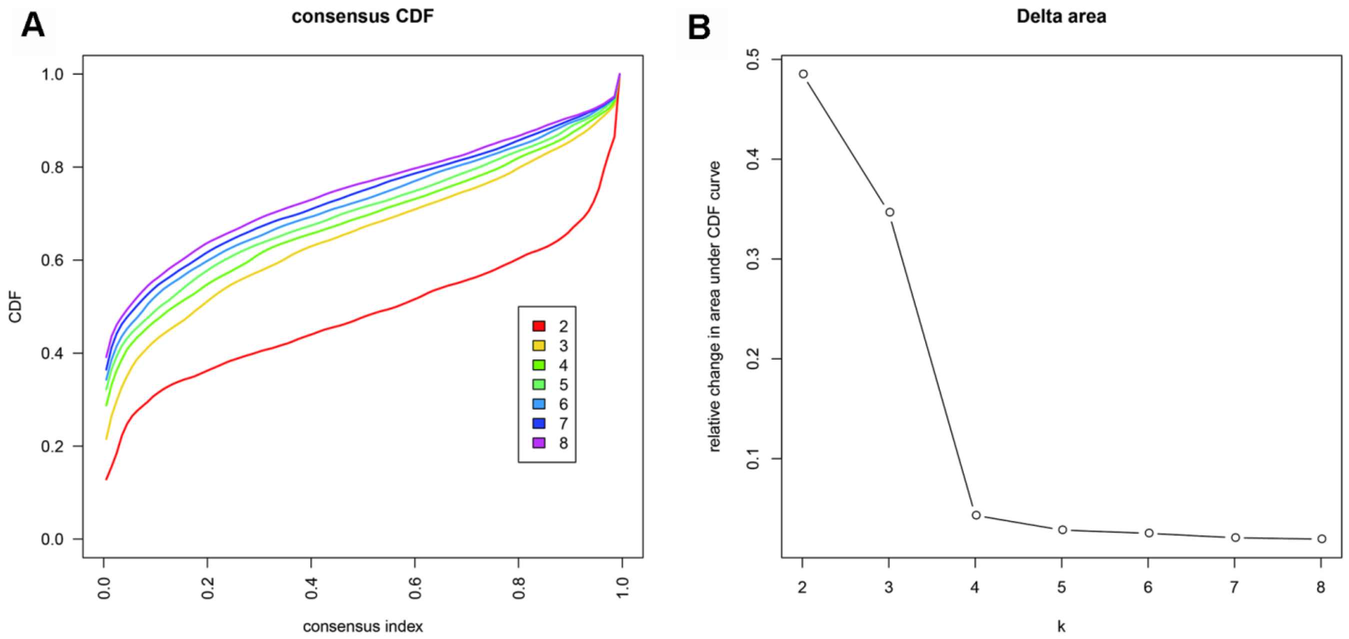

Supplementary Figure 2. Assessment of the optimal number of clusters. (**A**) The optimization process of Consensus Cumulative Distribution Function (CDF). The abscissa axis represents the consensus index; the ordinate axis represents the CDF value. (**B**) The change of K value in Delta area. The abscissa axis represents the change of K value, and the ordinate axis represents relative change in area under CDF curve.

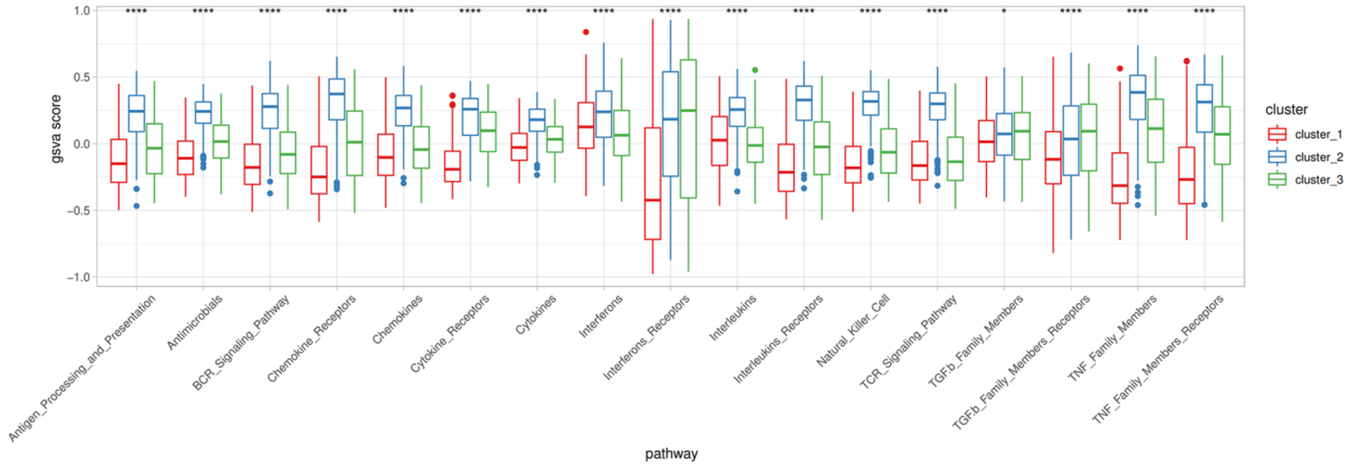

Supplementary Figure 3. The enrichment scores of immune-related pathways are significantly different in the three clusters: The x-axis is 17 different immune-related pathways, derived from ImmPort. The y-axis is the pathway enrichment score calculated based on the Gene Set Variation Analysis (GSVA) method. Perform Kruskal test in clusters (*, P <0.05; **, P <0.01; ***, P <0.001; ****, P <0.0001).

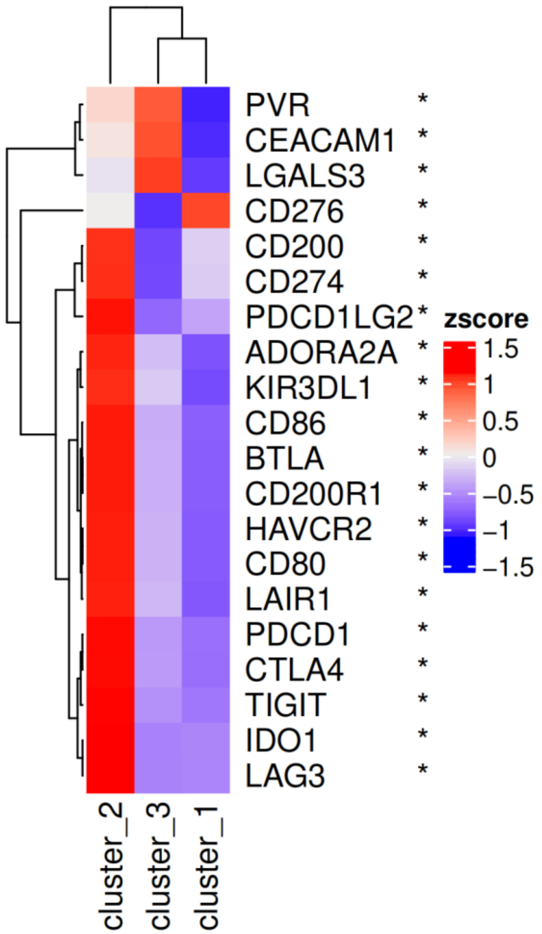

Supplementary Figure 4. The average expression of immune checkpoint genes in each cluster: Suppressive immune checkpoints are significantly different in the three clusters. The expression value is normalized by log2-transformed and zscore. * Indicates that there is a significant difference in gene expression between the three clusters (Kruskal test, *, P <0.05).

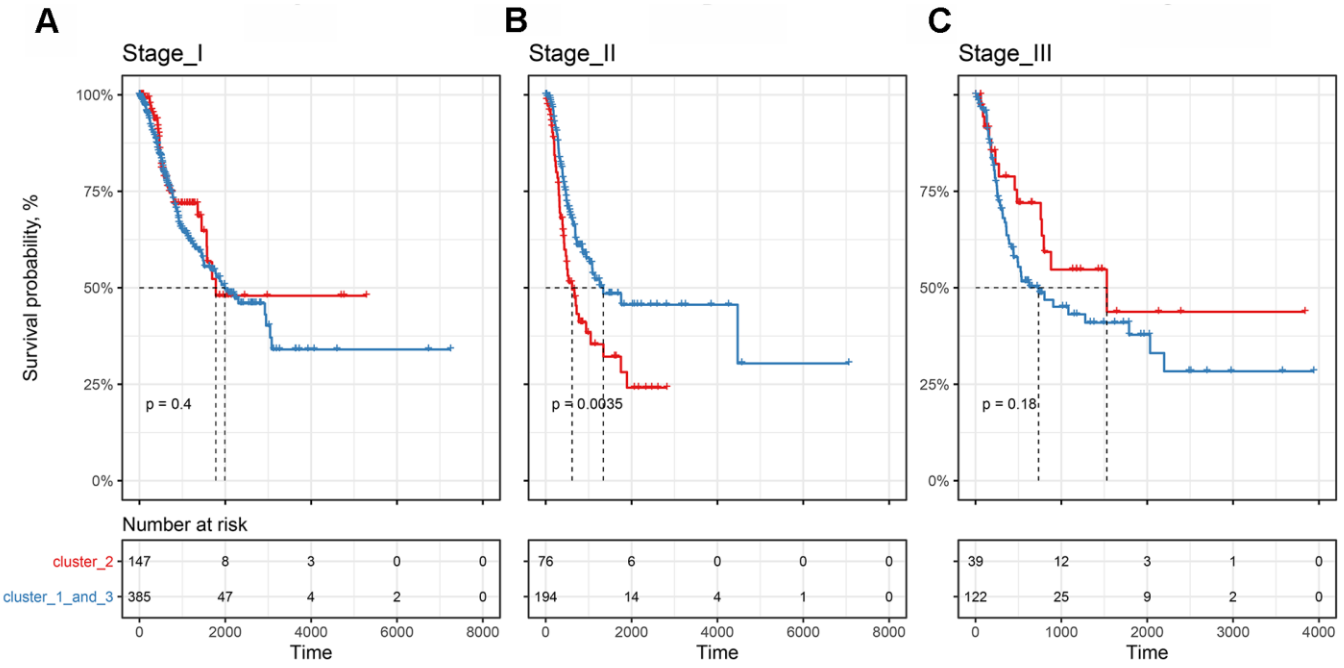

Supplementary Figure 5. Survival analysis in different clusters corresponding to different stages: Immunization subgroups can significantly distinguish the progression-free survival of Stage II NSCLC patients from others. The Stage IV sample size was too small, so it was deleted. The abscissa axis represents survival time, and the ordinate axis represents survival probability. The red curve represents cluster 2, the blue curve represents clusters _1 and_3. (**A**) Survival analysis curve of stage I NSCLC patients. (**B**) Survival analysis curve of stage II NSCLC patients. (**C**) Survival analysis curve of stage III NSCLC patients.

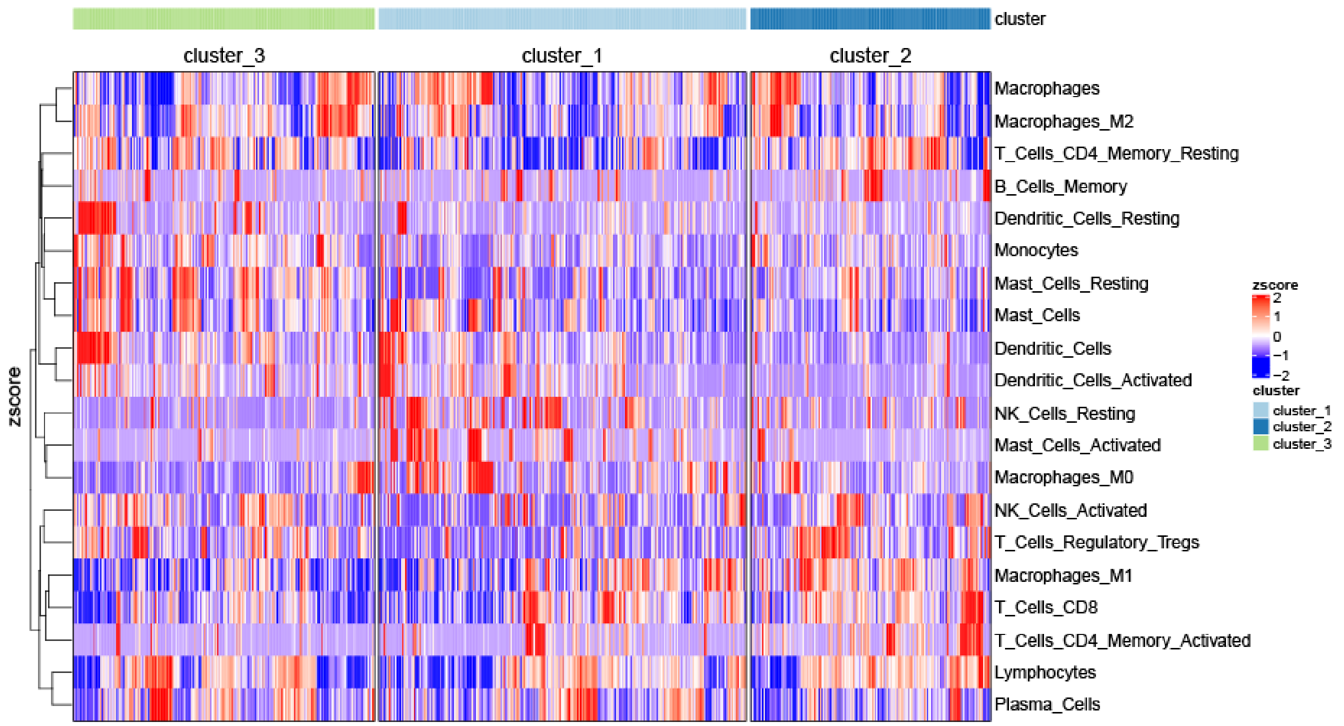

Supplementary Figure 6. Distribution of immune cell infiltration in different clusters.
